# Dance classification using pretrained deep learning models integrated with the circular Fermatean fuzzy MARCOS method

**DOI:** 10.1038/s41598-025-20808-x

**Published:** 2025-10-23

**Authors:** Yanru Wang

**Affiliations:** School of Art, Dance Studies, Wuhan Sports University, Wuhan, 430079 Hubei China

**Keywords:** Circular Fermatean fuzzy sets, Dance classification, Deep learning models, MARCOS approach, Multi-criteria decision-making, Engineering, Mathematics and computing

## Abstract

Pretrained deep learning models offer a method for classifying dance by analyzing movement behaviors in videos or using sensors to assess the degree of automation in recognizing different types of dances. These models utilize transfer learning strategies to enhance recognition accuracy across various datasets. This study presents a hybrid framework for identifying and classifying dance styles using pretrained deep learning models, evaluated under multiple performance criteria. To address the inherent uncertainty and complexity in selecting the best-performing models, a novel circular Fermatean fuzzy measurement of alternatives and ranking based on the compromise solution (CFF-MARCOS) approach is proposed. Unlike existing methods, the integration of circular Fermatean fuzzy sets (CFFS) into the MARCOS framework enables more refined handling of hesitation and ambiguity in expert evaluations. A case study involving ten pretrained models, seven criteria, and three experts demonstrates the superiority of the proposed method in generating robust and interpretable rankings. Results highlight improved decision reliability and clarity in model selection for automated dance classification tasks.

## Introduction

Pretrained deep learning models for dance classification represent a promising and impactful area of research in artificial intelligence, with real-world applications in entertainment, healthcare (e.g., rehabilitation), and robotics (e.g., motion planning and imitation learning). These models utilize computer vision and machine learning technologies to identify and tag various dance styles automatically. The growing availability of large-scale video datasets and significant advancements in pose estimation have accelerated this trend. Neural network architecture pre-trained on 2D image data, convolutional neural networks (CNNs)^1^, and recurrent neural networks^2^, are increasingly employed for extracting both spatial and temporal patterns in dance movements. Since these models are pretrained on vast datasets, they not only reduce training time but also enhance generalization in new tasks.

The cultural identification forms, consisting of dancing via role-based classification procedures, demand the ability to differentiate styles, e.g., ballet, hip-hop, salsa, and classical dancing, based on highly granular variances in the efficiency of the body-related movement, rhythm, and choreography. However, selecting the most suitable pretrained deep learning model for this classification task remains a non-trivial problem. This is especially true when performance must be evaluated across multiple criteria, such as classification accuracy, model complexity, training time, interpretability, latency, generalization ability, and computational cost. A singular focus on accuracy alone is insufficient for deployment-level decisions. Thus, the selection process is best framed as a multi-criteria decision-making (MCDM) problem, wherein decision-makers must balance several often-conflicting attributes to determine the optimal model.

The MCDM framework is widely employed in various domains for evaluating alternatives defined by both qualitative and quantitative measures. In the context of dance classification, MCDM enables professionals to integrate expert judgment with empirical performance to select the most balanced model. However, traditional MCDM methods typically assume deterministic or precise input data, making them inadequate for capturing the uncertainty, vagueness, and hesitation that characterize real-world expert evaluations. This limitation underscores the need for more expressive uncertainty modeling.

The paper employs one of the MCDM methods known as the MARCOS method. MARCOS, suggested by Stevi et al.^3^, is an efficient MCDM tool used to rank the alternatives under consideration, where ideally no thorough compromise should be made. Still, an ideal is achieved through an appropriate compromise-based ranking scheme. By considering both optimistic and pessimistic assessments, it provides more consistent rankings. Initially used to classify dances, the MARCOS method enables not only the ranking of trained models according to a single criterion but also across a complex set of performance-related features, making it highly suitable for addressing this multidimensional set of problems. Although it has evolved, even the existing fuzzy and hesitant fuzzy approaches are not ideal in representing high-order ambiguity and interdependencies in application to high subjective and dimensional domains. In dance classification, where opinion is highly divergent among experts as to matters of aesthetics and technical performance, the classical methods have been found poor in addressing such subtlety. Moreover, such techniques are not described as computationally scalable and are unreliable when combined with deep pretrained neural networks that can be used to classify images. To address them, the CFF-based MARCOS method proposed has higher expressiveness in the context of modelling uncertainty and feasibility in a real-life scenario. It is simple in structure and computationally efficient; it can be applied to tasks of higher dimensionality and can bring about the reliability and interpretability of MCDM results.

To test the strength of the potential model, a detailed comparison is conducted between the current MCDM techniques, i.e., WASPAS^4^, VIKOR^5^, EDAS^6^, COCOSO^7^, and TOPSIS^8^, in this research using terms like “high,” “medium,” and “low” to provide comparative performance measures. Additionally, sensitivity analysis is performed by varying the weights of decision-makers in different models to reassess the impact on model rankings. This way, the decision support system should be not only accurate but also consistent, even when expert opinions change.

Overall, this paper aims to develop a new decision support framework by combining the CFFS and the MARCOS method, designed to tackle the challenge of selecting the most suitable pretrained deep learning model for classifying dance, referred to as CFF-MARCOS. The methodology effectively characterizes uncertainty, illustrating specialist uncertainty decision tracks through three-dimensional unambiguous information and the comparative evaluation of alternatives based on compromise assessment. This proposed approach not only addresses a significant research gap but also demonstrates its effectiveness through a real-life case study, providing valuable theoretical, computational, and practical implications for decision-making in a specific, highly complex artificial intelligence application, such as dance classification. Abbreviation Section displays the list of abbreviations used in this paper.

### Research gap and motivation

Although pre-trained deep learning models have been applied in dance classification across various domains, such as heritage preservation, sports analytics, and virtual choreography, existing selection methods predominantly rely on single-objective criteria, like classification accuracy. Such limited evaluation metrics fail to address the inherently multi-dimensional nature of real-world decision-making, particularly in artistic and creative contexts where interpretability, generalization, and computational efficiency also hold critical value. While some studies have incorporated MCDM techniques to address model selection, they typically employ conventional or crisp fuzzy environments, which are inadequate in expressing the levels of vagueness, hesitation, and ambiguity often found in expert opinions. Moreover, although fuzzy set extensions have been used to improve uncertainty modeling, these approaches remain linear and cannot represent circular or angular uncertainty that better reflects human cognitive hesitation in subjective evaluations. No prior study has integrated CFFS into the MARCOS method for evaluating pretrained deep learning models in dance classification. This integration is not merely an application of existing frameworks but constitutes a theoretical advancement: the standard MARCOS procedure is extended to accommodate the geometric and structural properties of CFFS, particularly in normalization, utility function construction, and aggregation steps.

Therefore, this paper aims to fill both a methodological and application-oriented gap by proposing the CFF-MARCOS framework, an advanced decision-making model capable of robustly handling three-dimensional circular uncertainty while delivering compromise-based, interpretable, and stable rankings. This contributes both to the theory of fuzzy MCDM models and practical applications in AI-based dance classification.

### Objectives and contributions of the study

This study aims to develop a robust decision support framework for identifying the most appropriate pretrained deep learning model for dance classification. By integrating CFFS into the MARCOS methodology, the proposed approach better captures uncertainty in expert judgments while providing a reliable compromise solution. The primary contributions of this research include:

- An innovative application of the MARCOS method under a CFF environment for assessing pretrained models in dance classification.
- A formalized decision model incorporating both benefit and cost-type criteria, evaluated by a panel of expert decision-makers.
- A real-world case study demonstrating the superiority of the proposed CFF-MARCOS framework over traditional MCDM approaches, including WASPAS, VIKOR, EDAS, COCOSO, and TOPSIS.
- A comparative and sensitivity analysis highlighting the method’s flexibility, robustness, and practical significance for other intelligent classification problems.

### Layout of the study

The remaining paper is outlined as follows: Section "Literature review" summarizes the literature review conducted for this paper. Section "Preliminaries and research methodology" explains the fundamental concepts specific to CFFS and the proposed CCF-MARCOS methodology. Section "Case study: dance classification using pretrained deep learning models" employs this methodology to solve a case study on dance classification using pretrained deep learning models and discusses the results. Section 5 carries out comparative and sensitivity analyses that present practical management implications, challenges, and benefits. Finally, Section 6 concludes with significant findings and outlines future research directions.

## Literature review

The section presents a literature review of published studies related to the classification of dance using pretrained deep learning models and the incorporation of MCDM techniques in fuzzy settings. Various research efforts have utilized deep neural networks to accurately recognize dance forms, employing popular networks such as VGG, ResNet, and CNN-LSTM. Concurrently, MCDM techniques like MARCOS have been applied across numerous fields to address diverse situations requiring decisions to be made amidst uncertainty. However, few studies combine deep learning with dance classification while incorporating advanced fuzzy MCDM methods, particularly the CFF-MARCOS approach that this paper aims to highlight to fill the identified research gap.

### Dance classification using pretrained deep learning models

Recent studies have explored pretrained deep learning models for automatic classification of various dance types based on spatial and temporal features. For instance, Biswas et al.^9^ utilized a VGG-based model to classify Indian classical dance forms, while Jain et al.^10^ employed a modified ResNet50 to enhance classification performance. Gupta et al.^11^ developed a hybrid model combining CNN and SVM to identify ballet, hip-hop, and bhangra dances. Similarly, Rani and Devarakonda^12^ used a CNN-LSTM architecture to process video data for pose estimation and classification. Kritsis et al.^13^ and Qin and Meng^14^ proposed other studies using convolution-oriented models to assess the quality of dance motions. Kim^15^ suggests a Korean dance metadata-enhanced real-time recognition system. Even though these works report encouraging findings, they mostly manage human subjectivity or expert uncertainty through traditional measures of accuracy and fail to employ fuzzy sets as a method of deciding or making uncertain decisions. The present research builds on this body of knowledge through the utilization of CFF-MARCOS methodology to assess pretrained deep learning models by incorporating expert-based thresholds in an uncertain setting.

### Fuzzy sets in MCDM

Fuzzy sets have been used long since in the MCDM paradigm to represent uncertainty, vagueness, and imprecision present in actual decision-making. The concept of fuzzy set theory, first presented by Zadeh^16^, provides a method for articulating uncertainty, where the membership degree (MD) of an element is within the interval $[\text{0,1}]$. Nonetheless, fuzzy sets cannot discuss the difference in terms of a degree of non-membership (NMD). Consequently, the second type of fuzzy sets, intuitionistic fuzzy sets (IFSs), developed by Atanassov^17^, involves MD and NMD variations. Subsequently, Pythagorean fuzzy sets (PyFS) were proposed by Yager^18^, who allowed for additional freedom under the condition that the square sum of MD and NMD belonged to $[\text{0,1}]$. Fermatean fuzzy sets (FFS), introduced by Senapati^19^, represent another generalization where the properties MD and NMD have a cube sum that lies in the range $[\text{0,1}]$. Although this constitutes an improvement, these models remain linear and cannot account for circular or angular uncertainty in expert judgments. To address this limitation, CFFS^20^ has been proposed. These also take into account a radius value and MD and NMD, to better and didactically represent more mathematically how human preferences work, as well as experts’ uncertainty. CFFS gives a three-dimensional representation of uncertainty, and as such, it is well suited to general multi-dimensional decision-making problems, such as pretrained model selection in dance classification.

### MARCOS-based applications

MARCOS’ approach suggested by Stevi et al.^21^ is a new practical approach to the family of MCDM techniques. It has the utility weight of each alternative computed as a function of the reference values of the ideal and the anti-ideal solutions, which provides a higher and easier interpretation degree of rankings. The fact that it is simple to calculate and is also applicable across a wide spectrum makes its use possible in a broad range of fuzzy as well as hybrid worlds. An example is where Zhang et al.^22^ incorporated a Bayesian Best–Worst Method (BBWM) together with MARCOS to determine the regulatory risk of power grid enterprises. Memarpour Ghiaci et al.^23^ employed the use of MARCOS in a spherical fuzzy framework to increase the efficiency of emergency departments amid the COVID-19 pandemic challenge. On the same note, Wang and Dang^24^ hypothesized a fuzzy AHP-MARCOS mechanism of the third-party logistics selection in Industry 4.0. MARCOS have been generalized by other scholars to apply in complicated fuzzy structure, such as the Pythagorean hesitant fuzzy^25^, linear diophantine fuzzy^26^, and intuitionistic fuzzy^27^, to complement ambiguity and uncertainty in decision-making. Mishra et al.^28^ used an operator-centred MARCOS strategy to address circular supplier selection issues in Pythagorean fuzzy context, whereas Rong et al.^29^ also used a similar approach to distribute to logistic distribution centers in a cubic Fermatean fuzzy setting. These models address higher-order uncertainty but still exhibit structural or interpretive complexity. Recent studies such as^30–32^, and^33^ have further emphasized the need for efficient fuzzy MCDM integrations, especially in fields requiring nuanced evaluations. Despite these advancements, no prior study has integrated CFFS with the MARCOS method to assess deep learning models for dance classification. The proposed CFF-MARCOS approach contributes a novel solution capable of capturing circular hesitancy, enhancing model evaluation through structured compromise-based reasoning, and offering improved decision quality in high-uncertainty, creative domains.

## Preliminaries and research methodology

This section discusses the basic concept related to CFFS and proposes the CFF-MARCOS approach. Abbreviation section shows the list of symbols with their description.

### Definition 1:

Ref^19^. Consider a fixed universe $\dot{X}$ and its subset ${\text{\.{H}}}=\{x, {\mathfrak{p}}_{{\text{\.{H}}}} \left(x\right),{\mathfrak{q}}_{{\text{\.{H}}}}(x)| x\epsilon {\text{\.{X}}}\}$ be an FFS, where ${\mathfrak{p}}_{{\text{\.{H}}}} \left(x\right)\in [0, 1]$ represents MD, and ${\mathfrak{q}}_{{\text{\.{H}}}} \left(x\right)\in [0, 1]$ represents NMD. These degrees satisfy the following condition:

$$0 \le {\mathfrak{p}}_{\text{{\text{\.{H}}}}}^{3}\left(x\right)+{\mathfrak{q}}_{\text{{\text{\.{H}}}}}^{3}(x)\le 1.$$

Additionally, the HD for each FFS ${\text{\.{H}}}$ is defined as:

$${\mathcalligra{h}}_{\text{\.{H}}}=\sqrt[3]{1-{\mathfrak{p}}_{\text{\.{H}}}^{3}\left(x\right)-{\mathfrak{q}}_{\text{\.{H}}}^{3}(x)}.$$

### Definition 2:

Ref^20^. Consider a fixed universe $\dot{X}$ and its subset $\dot{H}= \{ x, {\mathfrak{p}}_{\dot{H}} \left(x\right),{\mathfrak{q}}_{\dot{H}}\left(x\right);{{\d{r}}}_{\dot{H}}\left(x\right)| x\epsilon \dot{X}\}$ be a CFFS, where ${\mathfrak{p}}_{\dot{H}} \left(x\right)\in [0, 1]$ represents MD,${\mathfrak{q}}_{\dot{H}} \left(x\right)\in [0, 1]$ represents NMD, and ${{\d{r}}}_{\dot{H}}\left(x\right)\in [0, 1]$ represents the radius term. These degrees satisfy the following condition:

$$0 \le {\mathfrak{p}}_{\dot{H}}^{3}\left(x\right)+{\mathfrak{q}}_{\dot{H}}^{3}(x)\le 1.$$

Additionally, the HD for each CFFS ${\text{\.{H}}}$ is defined as:

$${\mathcalligra{h}}_{\text{{\text{\.{H}}}}}=\sqrt[3]{1-{\mathfrak{p}}_{\dot{H}}^{3}\left(x\right)-{\mathfrak{q}}_{\dot{H}}^{3}(x)}.$$

### Circular Fermatean fuzzy MARCOS approach

**Fig. 1:**
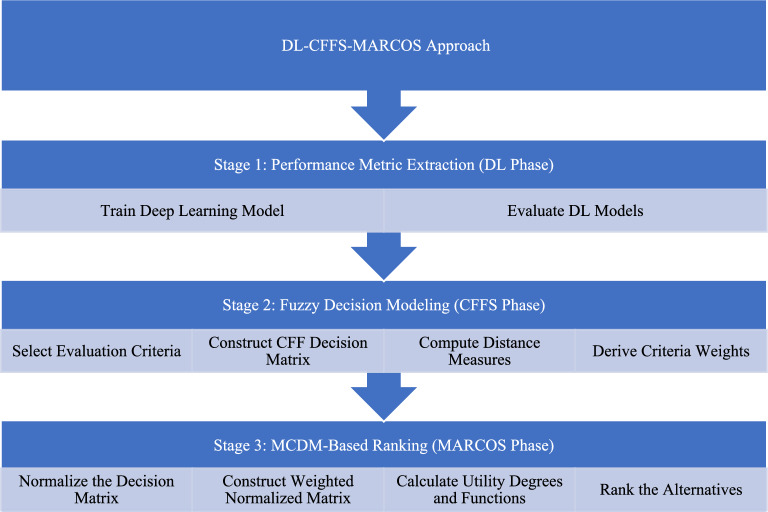
Flowchart of the DL-CFF-MARCOS approach.

A MCDM approach is employed to calculate the weight of each criterion using a weighted averaging model, specifically the CFFS and the circular Fermatean fuzzy weighted averaging (CFFWA) operator. Linguistic variables are converted into CFFVs to facilitate further analysis. To address the classification challenge, the MARCOS method is proposed, which is designed to evaluate and rank alternatives based on a compromise solution. Although the MARCOS approach is an effective decision-making tool, it cannot represent ambiguous or fuzzy information. To overcome this limitation, a fuzzy extension is incorporated to enhance its ability to handle uncertainty and imprecision in real-world scenarios. The explanation of the CFF-MARCOS technique will be presented as follows and illustrated in Fig. 1:

(i) $D= \{\text{1,2},. . . ,f \}$, denotes the set of decision makers (DMs) with weights represented by $\mathcalligra{w} = [ {\mathcalligra{w}}_{1}, {\mathcalligra{w}}_{2}, . . . , {\mathcalligra{w}}_{f}]$ and ${\sum }_{D=1}^{f}{\mathcalligra{w}}_{D} = 1$.

1 $${\mathcalligra{w}}_{D}=\frac{\left({\mathfrak{p}}_{D} + {\d{r}}_{D} . ( \frac{{\mathfrak{p}}_{D}}{{\mathfrak{p}}_{D}+{\mathfrak{q}}_{D}} )\right)}{\sum_{k=1}^{l}\left({\mathfrak{p}}_{D} + {\d{r}}_{D} . ( \frac{{\mathfrak{p}}_{D}}{{\mathfrak{p}}_{D}+{\mathfrak{q}}_{D}} )\right)}$$

(ii) There are ${C}_{\mu }$ criteria, and each has a weight of $\mathcal{W}=[{\zeta }_{1}, {\zeta }_{2},. . . , {\zeta }_{n}]$

$$\sum_{\mu =1}^{n}{\zeta }_{\mu }=1, (\mu =1, 2, . . . , n)$$

**Step 1.** Experts evaluate the criteria.

The linguistic evaluations for the criterion and DMs are shown in Table 1.

**Table 1:** Linguistic variables to evaluate criteria and decision makers.

Expression	CFFVs $(\mathfrak{p}, \mathfrak{q}, {\d{r}})$		
Very important (VI)	$0.88$	$0.65$	$0.97$
Important (I)	$0.78$	$0.55$	$0.83$
Medium (M)	$0.58$	$0.5$	$0.45$
Unimportant (UI)	$0.38$	$0.65$	$0.43$
Very unimportant (VU)	$0.28$	$0.75$	$0.11$

**Step 2.** Create aggregated CFF decision matrix.

Given a set of decision-makers, let $D={\left[{\mathcal{G}}_{\mu D}\right]}_{n*f}(D=\text{1,2},\dots ,f;\mu =\text{1,2},\dots ,n)$ be the CFF decision matrix. Here, ${\mathcal{G}}_{\text{me}}$ indicates the evaluation of ${d}^{t}h$ regarding DMs the ${j}^{th}$ criteria. ${\mathcal{G}}_{\mu D}$ is employed by CFFVs, and it could be described that ${\mathcal{G}}_{\mu D}=\left({\mathfrak{p}}_{{\mathcal{G}}_{\mu D}},{\mathfrak{q}}_{{\mathcal{G}}_{\mu D}},{{\d{r}}}_{{\mathcal{G}}_{\mu D}}\right)$.

The aggregated CFF decision matrix is represented as $\hat{R} = \left[ {{\hat{\mathcal{G}}}_{{\mu D}} } \right]_{{n*f}}$

2 $$\begin{aligned} {\hat{\mathcal{G}}}_{\mu } = & CFFWA_{\zeta } \left( {{\mathcal{G}}_{{\mu 1}} ,{\mathcal{G}}_{{\mu 2}} , \ldots ,{\mathcal{G}}_{{\mu D}} } \right) \\ = & \left( {\sqrt[3]{{1 - \prod\limits_{{D = 1}}^{f} {\left( {1 - {\mathfrak{p}}_{{{\mathcal{G}}_{{\mu D}} }}^{3} } \right)^{{\mathcalligra{w}_{D} }} } }},\prod\limits_{{D = 1}}^{f} {\left( {{\mathfrak{q}}_{{{\mathcal{G}}_{{\mu D}} }}^{{\left( k \right)}} } \right)^{{\mathcalligra{w}_{D} }} } ,\prod\limits_{{D = 1}}^{f} {\left( {\d{r}_{{{\mathcal{G}}_{{\mu D}} }}^{{\left( k \right)}} } \right)^{{\mathcalligra{w}_{D} }} } } \right) \\ \end{aligned}$$

where $${\hat{\mathcal{G}}}_{\mu } = \left( {{\mathfrak{p}}_{{{\hat{\mathcal{G}}}_{\mu } }} ,{\mathfrak{q}}_{{{\hat{\mathcal{G}}}_{\mu } }} ,\d{r} _{{{\hat{\mathcal{G}}}_{\mu } }} } \right)$$.

Table 2 is used to conduct the linguistic assessments of the alternatives.

**Table 2:** Linguistic factors for an alternative ranking system.

Expression	CFFVs $(\mathfrak{p}, \mathfrak{q}, {\d{r}})$		
Extremely good (EG)	$1.00$	$0.00$	$0.50$
Very very good (VVG)	$0.85$	$0.56$	$0.91$
Very good (VG)	$0.80$	$0.60$	$0.85$
Good (G)	$0.70$	$0.65$	$0.34$
Medium good (MG)	$0.60$	$0.55$	$0.44$
Fair (F)	$0.50$	$0.45$	$0.27$
Medium bad (MB)	$0.40$	$0.75$	$0.96$
Bad (B)	$0.30$	$0.85$	$0.43$
Very bad (VB)	$0.20$	$0.90$	$0.10$
Very very bad (VVB)	$0.10$	$0.95$	$0.70$

**Step 3.** Find the optimal solutions for CFF.

The optimal solutions for the CFF positive ideal solution (CFFPIS) are ${\tau }^{+}=(\text{1,0},0)$ and CFF negative ideal solution (CFFNIS) is ${\tau }^{-}=(\text{0,1},0)$. Although the max and min operators define CFFNIS and CFFPIS, the results are said to not significantly differ from one another.

**Step 4.** Determine the distance measures.

A fuzzy normalized Euclidean distance equation is used for determining the distance measure. ${\mathcal{D}}_{\mu }^{+}$ and ${\mathcal{D}}_{\mu }^{-}$ are utilized in these equations below to demonstrate positive and negative distance measures, respectively.

3 $${\mathcal{D}}_{\mu }^{ + } = \sqrt {\left( {{\mathfrak{p}}_{{{\hat{\mathcal{G}}}_{\mu } }} - \tau ^{ + } } \right)^{2} + \left( {{\mathfrak{q}}_{{{\hat{\mathcal{G}}}_{\mu } }} - \tau ^{ + } } \right)^{2} + \left( {{\d{r}}_{{{\hat{\mathcal{G}}}_{\mu } }} -\tau ^{ + } } \right)^{2} }$$

4 $${\mathcal{D}}_{\mu }^{ - } = \sqrt {\left( {{\mathfrak{p}}_{{{\hat{\mathcal{G}}}_{\mu } }} - \tau ^{ - } } \right)^{2} + \left( {{\mathfrak{q}}_{{{\hat{\mathcal{G}}}_{\mu } }} - \tau ^{ - } } \right)^{2} + \left( {{\d{r}}_{{{\hat{\mathcal{G}}}_{\mu } }} - \tau ^{ - } } \right)^{2} }$$

**Step 5.** Find the values of the closeness coefficient (CC).

$C{C}_{\mu }$ is the CC of the ${\mu }^{th}$ criterion, and it is defined utilizing CFFPIS ${\mathcal{D}}_{\mu }^{+}$ and CFFNIS ${\mathcal{D}}_{\mu }^{-}$ as follows:

5 $$C{C}_{\mu }=\frac{{\mathcal{D}}_{\mu }^{-}}{{\mathcal{D}}_{\mu }^{-}+{\mathcal{D}}_{\mu }^{+}}$$

**Step 6.** Find the weights of criteria and alternatives.

The CC values provide the relative relevance of each criterion. It is mentioned that the finalized weights are computed by applying normalization, and the total weights should equal $1$.

After Steps 5 and 6, we obtain a decision matrix $\breve{D}$.

$$\left[ {\begin{array}{*{20}c} {\overset{\lower0.5em\hbox{$\smash{\scriptscriptstyle\smile}$}}{\zeta } _{1} } & {\overset{\lower0.5em\hbox{$\smash{\scriptscriptstyle\smile}$}}{\zeta } _{2} } & \ldots & {\overset{\lower0.5em\hbox{$\smash{\scriptscriptstyle\smile}$}}{\zeta } _{n} } \\ \end{array} } \right]$$

$$\overset{\lower0.5em\hbox{$\smash{\scriptscriptstyle\smile}$}}{D} = \left[ {\begin{array}{*{20}c} {\overset{\lower0.5em\hbox{$\smash{\scriptscriptstyle\smile}$}}{x} _{{11}} } & {\overset{\lower0.5em\hbox{$\smash{\scriptscriptstyle\smile}$}}{x} _{{12}} } & \ldots & {\overset{\lower0.5em\hbox{$\smash{\scriptscriptstyle\smile}$}}{x} _{{1n}} } \\ {\overset{\lower0.5em\hbox{$\smash{\scriptscriptstyle\smile}$}}{x} _{{21}} } & {\overset{\lower0.5em\hbox{$\smash{\scriptscriptstyle\smile}$}}{x} _{{22}} } & \ldots & {\overset{\lower0.5em\hbox{$\smash{\scriptscriptstyle\smile}$}}{x} _{{2n}} } \\ \vdots & \vdots & \ddots & \vdots \\ {\overset{\lower0.5em\hbox{$\smash{\scriptscriptstyle\smile}$}}{x} _{{\mu 1}} } & {\overset{\lower0.5em\hbox{$\smash{\scriptscriptstyle\smile}$}}{x} _{{\mu 2}} } & \ldots & {\overset{\lower0.5em\hbox{$\smash{\scriptscriptstyle\smile}$}}{x} _{{\mu n}} } \\ \end{array} } \right]$$

**Step 7.** Create a decision matrix for CFF that is expanded.

An expanded decision matrix $\breve{E}$ is built by determining the anti-ideal $\text{A}I$ and ideal $I$ solution.

$$\left[ {\begin{array}{*{20}c} {\overset{\lower0.5em\hbox{$\smash{\scriptscriptstyle\smile}$}}{\zeta } _{1} } & {\overset{\lower0.5em\hbox{$\smash{\scriptscriptstyle\smile}$}}{\zeta } _{2} } & \ldots & {\overset{\lower0.5em\hbox{$\smash{\scriptscriptstyle\smile}$}}{\zeta } _{n} } \\ \end{array} } \right]$$

6 $$\overset{\lower0.5em\hbox{$\smash{\scriptscriptstyle\smile}$}}{E} = \begin{array}{*{20}c} {\overset{\lower0.5em\hbox{$\smash{\scriptscriptstyle\smile}$}}{\dot{H}} _{1} } \\ {\overset{\lower0.5em\hbox{$\smash{\scriptscriptstyle\smile}$}}{\dot{H}} _{2} } \\ \vdots \\ {\overset{\lower0.5em\hbox{$\smash{\scriptscriptstyle\smile}$}}{\dot{H}} _{\mu } } \\ {\dot{H}I} \\ I \\ \end{array} \left[ {\begin{array}{*{20}c} {\overset{\lower0.5em\hbox{$\smash{\scriptscriptstyle\smile}$}}{x} _{{11}} } & {\overset{\lower0.5em\hbox{$\smash{\scriptscriptstyle\smile}$}}{x} _{{12}} } & \ldots & {\overset{\lower0.5em\hbox{$\smash{\scriptscriptstyle\smile}$}}{x} _{{1n}} } \\ {\overset{\lower0.5em\hbox{$\smash{\scriptscriptstyle\smile}$}}{x} _{{21}} } & {\overset{\lower0.5em\hbox{$\smash{\scriptscriptstyle\smile}$}}{x} _{{22}} } & \ldots & {\overset{\lower0.5em\hbox{$\smash{\scriptscriptstyle\smile}$}}{x} _{{2n}} } \\ \vdots & \vdots & \ddots & \vdots \\ {\overset{\lower0.5em\hbox{$\smash{\scriptscriptstyle\smile}$}}{x} _{{\mu 1}} } & {\overset{\lower0.5em\hbox{$\smash{\scriptscriptstyle\smile}$}}{x} _{{\mu 2}} } & \ldots & {\overset{\lower0.5em\hbox{$\smash{\scriptscriptstyle\smile}$}}{x} _{{\mu n}} } \\ {\overset{\lower0.5em\hbox{$\smash{\scriptscriptstyle\smile}$}}{x} _{{ai1}} } & {\overset{\lower0.5em\hbox{$\smash{\scriptscriptstyle\smile}$}}{x} _{{ai2}} } & \ldots & {\overset{\lower0.5em\hbox{$\smash{\scriptscriptstyle\smile}$}}{x} _{{ain}} } \\ {\overset{\lower0.5em\hbox{$\smash{\scriptscriptstyle\smile}$}}{x} _{{id1}} } & {\overset{\lower0.5em\hbox{$\smash{\scriptscriptstyle\smile}$}}{x} _{{id2}} } & \ldots & {\overset{\lower0.5em\hbox{$\smash{\scriptscriptstyle\smile}$}}{x} _{{idn}} } \\ \end{array} } \right]$$

The $AI$ is the worst alternative, whereas the $I$ is an alternative with the most acceptable performance. They are determined by employing Eqs. (7) and (8), respectively.

7 $$AI = \mathop {{\text{min}}}\limits_{i} \overset{\lower0.5em\hbox{$\smash{\scriptscriptstyle\smile}$}}{x} _{{ij}} {\text{, if }}j \in B{\text{ and }}\mathop {{\text{max}}}\limits_{i} \overset{\lower0.5em\hbox{$\smash{\scriptscriptstyle\smile}$}}{x} _{{ij}} {\text{, if }}j \in C$$

8 $$I = \mathop {{\text{max}}}\limits_{i} \tilde{x}_{{ij}} {\text{, if }}j \in B{\text{ and }}\mathop {{\text{min}}}\limits_{i} \overset{\lower0.5em\hbox{$\smash{\scriptscriptstyle\smile}$}}{x} _{{ij}} {\text{, if }}j \in C$$

It is clear that $B$ consists of benefit-type criteria, while $C$ consists of cost-type criteria.

**Step 8.** Construct the decision matrix for the normalized CFF.

Following is the process of obtaining the normalized value ${\breve{{\xi }}}_{ij}$ of the alternatives.

9 $$\overset{\lower0.5em\hbox{$\smash{\scriptscriptstyle\smile}$}}{\xi } _{{ij}} = \left\{ {\begin{array}{*{20}l} {\frac{{\overset{\lower0.5em\hbox{$\smash{\scriptscriptstyle\smile}$}}{x} _{{aij}} }}{{\hat{x}_{{idj}} }},j \in B} \hfill \\ {\frac{{\overset{\lower0.5em\hbox{$\smash{\scriptscriptstyle\smile}$}}{x} _{{idj}} }}{{\hat{x}_{{aij}} }},j \in C} \hfill \\ \end{array} } \right.$$

**Step 9.** Construct the weighted CFF decision matrix.

The weighted values for each alternative are obtained as follows.

10 $${}^{{''}}\overset{\lower0.5em\hbox{$\smash{\scriptscriptstyle\smile}$}}{\Omega } _{{ij}} = \overset{\lower0.5em\hbox{$\smash{\scriptscriptstyle\smile}$}}{\xi } _{{ij}} {\kern 1pt}{\overset{\lower0.5em\hbox{$\smash{\scriptscriptstyle\smile}$}}{\mathcal{W}} }_{j}$$

${\breve{{\zeta }}}_{j}$ in Eq. (10) denotes the ${j}^{th}$ criterion’s relative relevance.

**Step 10.** Construct the ${\breve{{\d{{\text{\.{S}}}}}}_{i}}$ matrix.

To obtain the values of ${\breve{{\d{{\text{\.{S}}}}}}_{i}}$ matrix, Eq. (11) is applied.

11 $$\overset{\lower0.5em\hbox{$\smash{\scriptscriptstyle\smile}$}}{\d{\text{\.{S}}} } _{i} \sum\nolimits_{{i = 1}}^{n} {{}^{{''}}\overset{\lower0.5em\hbox{$\smash{\scriptscriptstyle\smile}$}}{\Omega } _{{ij}} }$$

**Step 11.** Determine the utility degrees of alternatives.

The utility degrees of alternatives are determined through Eqs. (12) and (13).

12 $${\overset{\lower0.5em\hbox{$\smash{\scriptscriptstyle\smile}$}}{\mathfrak{K}} }_{I}^{ - } = \frac{\overset{\lower0.5em\hbox{$\smash{\scriptscriptstyle\smile}$}}{\d{\text{\.{S}}} } _{i}}{\overset{\lower0.5em\hbox{$\smash{\scriptscriptstyle\smile}$}}{\d{\text{\.{S}}} } _{ai}}$$

13 $${\overset{\lower0.5em\hbox{$\smash{\scriptscriptstyle\smile}$}}{\mathfrak{K}} }_{I}^{+} = \frac{\overset{\lower0.5em\hbox{$\smash{\scriptscriptstyle\smile}$}}{\d{\text{\.{S}}} } _{i}}{\overset{\lower0.5em\hbox{$\smash{\scriptscriptstyle\smile}$}}{\d{\text{\.{S}}} } _{id}}$$

**Step 12.** Identify the utility function of alternatives.

By using Eq. (14), the utility functions of alternatives are computed.

14 $$\begin{array}{*{20}c} {f\left( {{\mathfrak{K}}_{i} } \right) = \frac{{{\overset{\lower0.5em\hbox{$\smash{\scriptscriptstyle\smile}$}}{\mathfrak{K}} }_{i}^{ + } + {\overset{\lower0.5em\hbox{$\smash{\scriptscriptstyle\smile}$}}{\mathfrak{K}} }_{i}^{ - } }}{{1 + \frac{{1 - f\left( {{\overset{\lower0.5em\hbox{$\smash{\scriptscriptstyle\smile}$}}{\mathfrak{K}} }_{i}^{ + } } \right)}}{{f\left( {{\overset{\lower0.5em\hbox{$\smash{\scriptscriptstyle\smile}$}}{\mathfrak{K}} }_{i}^{ + } } \right)}} + \frac{{1 - f\left( {{\overset{\lower0.5em\hbox{$\smash{\scriptscriptstyle\smile}$}}{\mathfrak{K}} }_{i}^{ - } } \right)}}{{f\left( {{\overset{\lower0.5em\hbox{$\smash{\scriptscriptstyle\smile}$}}{\mathfrak{K}} }_{i}^{ - } } \right)}}}}} \\ \end{array}$$

In Eq. (14), $f\left({\breve{{\mathfrak{K}}}}_{i}^{+}\right)$ represents the utility function of the ideal solution, whereas $f\left({\widetilde{\mathfrak{K}}}_{i}^{-}\right)$ represents the utility function as per the anti-ideal solution. $f\left({\breve{{\mathfrak{K}}}}_{i}^{+}\right)$ and $f\left({\breve{{\mathfrak{K}}}}_{i}^{-}\right)$ are calculated using Eqs. (15) and (16).

15 $$\begin{array}{*{20}c} {f\left( {{\overset{\lower0.5em\hbox{$\smash{\scriptscriptstyle\smile}$}}{\mathfrak{K}} }_{i}^{ + } } \right) = \frac{{{\overset{\lower0.5em\hbox{$\smash{\scriptscriptstyle\smile}$}}{\mathfrak{K}} }_{i}^{ - } }}{{{\overset{\lower0.5em\hbox{$\smash{\scriptscriptstyle\smile}$}}{\mathfrak{K}} }_{i}^{ - } + {\overset{\lower0.5em\hbox{$\smash{\scriptscriptstyle\smile}$}}{\mathfrak{K}} }_{i}^{ + } }}} \\ \end{array}$$

16 $$\begin{array}{*{20}c} {f\left( {{\overset{\lower0.5em\hbox{$\smash{\scriptscriptstyle\smile}$}}{\mathfrak{K}} }_{i}^{ - } } \right) = \frac{{{\overset{\lower0.5em\hbox{$\smash{\scriptscriptstyle\smile}$}}{\mathfrak{K}} }_{i}^{ + } }}{{{\overset{\lower0.5em\hbox{$\smash{\scriptscriptstyle\smile}$}}{\mathfrak{K}} }_{i}^{ - } + {\overset{\lower0.5em\hbox{$\smash{\scriptscriptstyle\smile}$}}{\mathfrak{K}} }_{i}^{ + } }}} \\ \end{array}$$

**Step 13.** Rank the alternatives.

## Case study: dance classification using pretrained deep learning models

The dance classification is a new area of computer vision that seeks to model the process of automatically identifying and classifying different types of dances based on image or video data. It involves extracting features related to movement patterns, body postures, and visual cues that characterize various dance styles, such as ballet, hip-hop, salsa, or classical. CNNs and deep learning have proven highly effective in recent years at recognizing these intricate spatiotemporal patterns. A powerful option for such tasks is a pretrained deep learning model, which is trained on a large corpus, like ImageNet, and can generalize effectively to sample data in specialized domains. Models such as VGG16, ResNet50, InceptionV3, and MobileNetV2 are known for their short training times and relatively low computational costs, while also delivering good performance, making them suitable for the dance classification task. To ensure consistency and realism, each model was assumed to be fine-tuned using standard deep learning parameters: an input size of $224\times 224\times 3$, batch size of $32, 50$ training epochs, and the Adam optimizer with a learning rate of $0.001$. These settings are commonly used in visual classification tasks and ensure comparability across architectures.

To select the most suitable pretrained model for a specific task, such as dance classification, it is essential to compare several performance indicators, including accuracy, precision, training time, model complexity, and more. This situation of MCDM requires a systematic analysis to evaluate all competing models using various conflicting criteria. The CFF-MARCOS approach is utilized in this study to address this challenge. It allows for integrating expert opinions in uncertain situations and efficiently ranking alternatives based on benefit and cost considerations. Its practicality lies in using swing vectors, which enhance the expressiveness of circular fuzzy sets when addressing imprecise expert judgments, making this framework more robust in real-life scenarios.

Here, the methodology illustrated in CFF-MARCOS will be applied in the case study to compare seven pretrained deep learning models (alternatives) for classifying dances using this modality. The choice of scheme relies on evaluations from three specialists in the fields (decision-makers) based on seven criteria, which include two cost-related and five benefit-related aspects. It is important to note that the dataset used in this case study was constructed hypothetically to demonstrate the utility of the proposed methodology. While no real video data was used, the decision matrix reflects plausible expert-based evaluations that simulate real-world performance outcomes. The seven evaluation criteria for the deep learning models are as follows:

- ${{\varvec{C}}}_{1}$**: Classification Accuracy:** This metric guarantees the general correctness of the model’s predictions by giving us the ratio between correctly predicted styles of dancing and the total number of samples. The greater the precision, the more impressive the cumulative result and stability of this model for dance classification.
- ${{\varvec{C}}}_{2}$**: Precision:** Precision is the percentage of correct predictions given a favorable outcome compared to the model’s total number of accurate predictions. The main advantage of high precision in dance recognition can be seen in the model, which can easily classify the type of dance, but with little false recognition.
- ${{\varvec{C}}}_{3}$**: F1-Score:** The F1-score is a harmonic mean of precision and recall that gives a balanced measure of a model’s capability to evade false positives and false negatives. It works particularly well when dance classes are imbalanced or analogous in their design.
- ${{\varvec{C}}}_{4}$**: Training Time:** This criterion is the time it takes to train with the set (seconds). Smaller training time models are computer efficient and are desired in environments where resources are limited or time is an important factor.
- ${{\varvec{C}}}_{5}$**: Inference Time per Frame:** Inference time (per frame) demonstrates the speed at which a model trains a single video or image frame in a particular sequence of real-time classification. The models with less inference time are the most appropriate in live dance recognition systems, where time is of the essence.
- ${{\varvec{C}}}_{6}$**: Model Complexity:** Model complexity is measured by the number of overall trainable parameters in the architecture. Less complex models with a smaller number of parameters require less memory and computation and are more appropriate for implementation on mobile or embedded systems.
- ${{\varvec{C}}}_{7}$**: Generalization Capability:** The generalization ability measures the effectiveness of a model with new and unseen data sets of style of dances. Gurus put it to the test with regard to differences in validation and strength. The larger the capacity, the less overfitting there would be and the more flexible the model.

These criteria were chosen due to the expert opinion regarding a thorough evaluation of classification models. These metrics are standard in measuring deep learning activities, covering both the quality of prediction and the efficiency. Moreover, these requirements were selected to conform to the technical limitations and standards of quality in the actual implementation of deep learning in visual recognition processes. Using this complex manner of decision making makes the decisions more reliable because the considerations used in the model are based on real-life practices.

In the study, these seven alternatives are employed:

- $${A}_{1}$$: VGG16
- $${A}_{2}$$: ResNet50
- $${A}_{3}:$$ InceptionV3
- $${A}_{4}$$: MobileNetV2
- $${A}_{5}$$: DenseNet121
- $${A}_{6}$$: Xception
- $${A}_{7}$$: EfficientNetB0

**Steps 1 and 2:** Instead of using raw classification metrics from deep learning models, a panel of domain experts assessed each model based on seven performance-related criteria (e.g., accuracy, precision, recall). The evaluations were performed with the predetermined terminology of these lingual terms, very good, fair and bad and translated into CFFVs as depicted in abbreviations section. This conversion enables the model to incorporate uncertainty and human subjectivity effectively. The resulting CFFVs constitute the decision matrix used in the CFF-MARCOS methodology. The decision-makers evaluated the importance of the criteria using the linguistic scale in Table 1 and rated the ten alternatives using the scale provided in Table 2. Tables 3 and 4 present the aggregated linguistic assessments for criteria and alternatives, respectively.

**Table 3:** Linguistic assessments for the rating of the evaluation criteria.

	$D{M}_{1}$	$D{M}_{2}$	$D{M}_{3}$
${C}_{1}$	$VI$	$M$	$I$
${C}_{2}$	$I$	$M$	$I$
${C}_{3}$	$M$	$UI$	$VI$
${C}_{4}$	$UI$	$UI$	$VI$
${C}_{5}$	$VU$	$I$	$M$
${C}_{6}$	$VU$	$I$	$M$
${C}_{7}$	$VU$	$I$	$M$

**Table 4:** Linguistic assessments for the rating of the alternatives.

		${{\varvec{C}}}_{1}$	${{\varvec{C}}}_{2}$	${{\varvec{C}}}_{3}$	${{\varvec{C}}}_{4}$	${{\varvec{C}}}_{5}$	${{\varvec{C}}}_{6}$	${{\varvec{C}}}_{7}$
${{\varvec{A}}}_{1}$	$D{M}_{1}$	$G$	$F$	$MG$	$VB$	$VVG$	$EG$	$B$
	$D{M}_{2}$	$G$	$F$	$MG$	$VB$	$VG$	$B$	$B$
	$D{M}_{3}$	$MG$	$F$	$MG$	$B$	$VG$	$B$	$B$
${{\varvec{A}}}_{2}$	$D{M}_{1}$	$B$	$MG$	$F$	$G$	$VG$	$B$	$B$
	$D{M}_{2}$	$B$	$B$	$F$	$G$	$F$	$VVG$	$VVB$
	$D{M}_{3}$	$B$	$B$	$F$	$G$	$F$	$VVG$	$VVB$
${{\varvec{A}}}_{3}$	$D{M}_{1}$	$VVG$	$B$	$F$	$MB$	$MB$	$VVG$	$VB$
	$D{M}_{2}$	$VVG$	$EG$	$B$	$MB$	$MB$	$G$	$VB$
	$D{M}_{3}$	$F$	$VVG$	$B$	$MB$	$B$	$G$	$VB$
${{\varvec{A}}}_{4}$	$D{M}_{1}$	$F$	$VG$	$B$	$MG$	$B$	$G$	$EG$
	$D{M}_{2}$	$F$	$VG$	$VVB$	$MG$	$B$	$G$	$B$
	$D{M}_{3}$	$MB$	$G$	$VVB$	$F$	$B$	$B$	$B$
${{\varvec{A}}}_{5}$	$D{M}_{1}$	$MB$	$G$	$VVG$	$F$	$B$	$B$	$B$
	$D{M}_{2}$	$MG$	$G$	$VVG$	$F$	$G$	$B$	$F$
	$D{M}_{3}$	$MG$	$MB$	$F$	$G$	$G$	$B$	$F$
${{\varvec{A}}}_{6}$	$D{M}_{1}$	$MG$	$MB$	$F$	$VVG$	$G$	$F$	$F$
	$D{M}_{2}$	$VVB$	$MB$	$F$	$G$	$F$	$F$	$G$
	$D{M}_{3}$	$VVB$	$B$	$G$	$VVB$	$MG$	$F$	$G$
${{\varvec{A}}}_{7}$	$D{M}_{1}$	$VVB$	$B$	$G$	$VVB$	$MG$	$F$	$G$
	$D{M}_{2}$	$VVG$	$B$	$G$	$VVB$	$MB$	$F$	$MB$
	$D{M}_{3}$	$B$	$F$	$G$	$B$	$MB$	$F$	$MG$

**Step 3.** Table 1 clarifies what the committee considers the ratings of the DMs. The weight of each DM is then retrieved with the help of Eq. (1). Table 5 contains their weights.

**Table 5:** Decision-makers’ weights.

	$D{M}_{1}$	$D{M}_{2}$	$D{M}_{3}$
Linguistic variables	$VI$	$M$	$M$
Weight	$0.462$	$0.264$	$0.264$

**Step 4.** Equation (2) is used to derive the aggregation CFF decision table in Table 6.

**Table 6:** Aggregated CFF decision matrix.

	$\mathfrak{p}$	$\mathfrak{q}$	${\d{r}}$
${C}_{1}$	$0.809$	$0.583$	$0.760$
${C}_{2}$	$0.742$	$0.540$	$0.708$
${C}_{3}$	$0.699$	$0.578$	$0.549$
${C}_{4}$	$0.662$	$0.653$	$0.538$
${C}_{5}$	$0.596$	$0.623$	$0.278$
${C}_{6}$	$0.596$	$0.623$	$0.278$
${C}_{7}$	$0.596$	$0.623$	$0.278$

**Step 5.** Following Table 6 and the equations below (3) to (5), Table 7 shows the output of calculating ${\mathcal{D}}_{\mu }^{+},{\mathcal{D}}_{\mu }^{-}$ and the CFF weights of each criterion. An example is the CFFPIS and CFFNIS, which are, respectively ${\tau }^{+}=(\text{1,0},0)$ and ${\tau }^{-}=(\text{0,1},0)$.

**Table 7:** The weights of criteria.

	${\mathcal{D}}_{\mu }^{+}$	${\mathcal{D}}_{\mu }^{-}$	*CC*	Normalized weights
${C}_{1}$	$0.977$	$1.186$	$0.548$	$0.152$
${C}_{2}$	$0.927$	$1.124$	$0.548$	$0.152$
${C}_{3}$	$0.853$	$0.984$	$0.536$	$0.149$
${C}_{4}$	$0.911$	$0.921$	$0.503$	$0.140$
${C}_{5}$	$0.793$	$0.758$	$0.489$	$0.136$
${C}_{6}$	$0.793$	$0.758$	$0.489$	$0.136$
${C}_{7}$	$0.793$	$0.758$	$0.489$	$0.136$

**Step 6.** Similarly, as in step 4, in this step, the values $\mathfrak{p},$ $\mathfrak{q}$ and ${\d{r}}$ are derived, first of all, using Table 2. As Table 8 shows, these values are as follows. Second, Table 8 and Eqs were applied. (3)-(5), Table 9 resulted.

**Table 8:** Aggregated $\mathfrak{p},\mathfrak{q}$, and ${\d{r}}$ values of alternatives as per each criterion.

	$\mathfrak{p}$	$\mathfrak{q}$	${\d{r}}$	$\mathfrak{p}$	$\mathfrak{q}$	${\d{r}}$	$\mathfrak{p}$	$\mathfrak{q}$	${\d{r}}$	$\mathfrak{p}$	$\mathfrak{q}$	${\d{r}}$
	${C}_{1}$			${C}_{2}$			${C}_{3}$			${C}_{4}$		
${A}_{1}$	$0.676$	$0.625$	$0.368$	$0.498$	$0.454$	$0.274$	$0.598$	$0.554$	$0.444$	$0.235$	$0.888$	$0.151$
${A}_{2}$	$0.299$	$0.851$	$0.434$	$0.492$	$0.696$	$0.439$	$0.498$	$0.454$	$0.274$	$0.698$	$0.653$	$0.344$
${A}_{3}$	$0.802$	$0.532$	$0.661$	$1.000$	$0.000$	$0.550$	$0.418$	$0.635$	$0.350$	$0.399$	$0.752$	$0.960$
${A}_{4}$	$0.572$	$0.500$	$0.291$	$0.346$	$0.529$	$0.641$	$0.236$	$0.903$	$0.561$	$0.576$	$0.525$	$0.390$
${A}_{5}$	$0.651$	$0.598$	$0.394$	$0.346$	$0.529$	$0.641$	$0.456$	$0.252$	$0.405$	$0.572$	$0.500$	$0.291$
${A}_{6}$	$0.474$	$0.739$	$0.567$	$0.377$	$0.778$	$0.777$	$0.572$	$0.500$	$0.291$	$0.541$	$0.464$	$0.361$
${A}_{7}$	$0.227$	$0.709$	$0.533$	$0.377$	$0.720$	$0.384$	$0.698$	$0.653$	$0.344$	$0.199$	$0.923$	$0.618$
	${C}_{5}$			${C}_{6}$			${C}_{7}$			–		
${A}_{1}$	$0.824$	$0.584$	$0.879$	$1.000$	$0.000$	$0.465$	$0.299$	$0.851$	$0.434$	–	–	–
${A}_{2}$	$0.408$	$0.312$	$0.439$	$0.738$	$0.683$	$0.644$	$0.236$	$0.903$	$0.561$	–	–	–
${A}_{3}$	$0.377$	$0.778$	$0.777$	$0.785$	$0.610$	$0.542$	$0.199$	$0.901$	$0.102$	–	–	–
${A}_{4}$	$0.299$	$0.851$	$0.434$	$0.645$	$0.701$	$0.366$	$1.000$	$0.000$	$0.465$	–	–	–
${A}_{5}$	$0.593$	$0.739$	$0.383$	$0.299$	$0.851$	$0.434$	$0.430$	$0.609$	$0.339$	–	–	–
${A}_{6}$	$0.634$	$0.567$	$0.346$	$0.498$	$0.454$	$0.274$	$0.627$	$0.551$	$0.309$	–	–	–
${A}_{7}$	$0.515$	$0.652$	$0.670$	$0.498$	$0.454$	$0.274$	$0.622$	$0.649$	$0.484$	–	–	–

**Table 9:** Aggregated CFF decision matrix for alternatives.

	${\mathcal{D}}_{\mu }^{+}$	${\mathcal{D}}_{\mu }^{-}$	CC	${\mathcal{D}}_{\mu }^{+}$	${\mathcal{D}}_{\mu }^{-}$	CC	${\mathcal{D}}_{\mu }^{+}$	${\mathcal{D}}_{\mu }^{-}$	CC	${\mathcal{D}}_{\mu }^{+}$	${\mathcal{D}}_{\mu }^{-}$	CC
	${C}_{1}$			${C}_{2}$			${C}_{3}$			${C}_{4}$		
${A}_{1}$	$0.794$	$0.856$	$0.519$	$0.730$	$0.788$	$0.519$	$0.815$	$0.868$	$0.516$	$1.181$	$0.301$	$0.203$
${A}_{2}$	$1.185$	$0.547$	$0.316$	$0.967$	$0.726$	$0.429$	$0.730$	$0.788$	$0.519$	$0.797$	$0.852$	$0.517$
${A}_{3}$	$0.871$	$1.140$	$0.567$	$0.550$	$1.517$	$0.734$	$0.929$	$0.656$	$0.414$	$1.360$	$1.069$	$0.440$
${A}_{4}$	$0.719$	$0.814$	$0.531$	$1.058$	$0.867$	$0.450$	$1.309$	$0.616$	$0.320$	$0.780$	$0.842$	$0.519$
${A}_{5}$	$0.797$	$0.860$	$0.519$	$1.058$	$0.867$	$0.450$	$0.724$	$0.965$	$0.571$	$0.719$	$0.814$	$0.531$
${A}_{6}$	$1.069$	$0.784$	$0.423$	$1.263$	$0.892$	$0.414$	$0.719$	$0.814$	$0.531$	$0.746$	$0.843$	$0.531$
${A}_{7}$	$1.176$	$0.648$	$0.355$	$1.026$	$0.607$	$0.371$	$0.797$	$0.852$	$0.517$	$1.369$	$0.654$	$0.323$
	${C}_{5}$			${C}_{6}$			${C}_{7}$			–		
${A}_{1}$	$1.070$	$1.274$	$0.544$	$0.465$	$1.489$	$0.762$	$1.185$	$0.547$	$0.316$	–	–	–
${A}_{2}$	$0.800$	$0.912$	$0.533$	$0.975$	$1.030$	$0.514$	$1.309$	$0.616$	$0.320$	–	–	–
${A}_{3}$	$1.263$	$0.892$	$0.414$	$0.843$	$1.031$	$0.550$	$1.210$	$0.245$	$0.168$	–	–	–
${A}_{4}$	$1.185$	$0.547$	$0.316$	$0.867$	$0.799$	$0.480$	$0.465$	$1.489$	$0.762$	–	–	–
${A}_{5}$	$0.927$	$0.753$	$0.448$	$1.185$	$0.547$	$0.316$	$0.900$	$0.673$	$0.428$	–	–	–
${A}_{6}$	$0.759$	$0.842$	$0.526$	$0.730$	$0.788$	$0.519$	$0.734$	$0.831$	$0.531$	–	–	–
${A}_{7}$	$1.053$	$0.914$	$0.465$	$0.730$	$0.788$	$0.519$	$0.893$	$0.863$	$0.491$	–	–	–

**Step 7.** We form an extended decision matrix by using the values of the CC (Table 10) stated in Eqs. (7) and (8). Thus, Table 10 is the formation of an extended decision matrix.

**Table 10:** Extended CFF decision matrix.

	${C}_{1}$	${C}_{2}$	${C}_{3}$	${C}_{4}$	${C}_{5}$	${C}_{6}$	${C}_{7}$
${A}_{1}$	$0.519$	$0.519$	$0.516$	$0.203$	$0.544$	$0.762$	$0.316$
${A}_{2}$	$0.316$	$0.429$	$0.519$	$0.517$	$0.533$	$0.514$	$0.320$
${A}_{3}$	$0.567$	$0.734$	$0.414$	$0.440$	$0.414$	$0.550$	$0.168$
${A}_{4}$	$0.531$	$0.450$	$0.320$	$0.519$	$0.316$	$0.480$	$0.762$
${A}_{5}$	$0.519$	$0.450$	$0.571$	$0.531$	$0.448$	$0.316$	$0.428$
${A}_{6}$	$0.423$	$0.414$	$0.531$	$0.531$	$0.526$	$0.519$	$0.531$
${A}_{7}$	$0.355$	$0.371$	$0.517$	$0.323$	$0.465$	$0.519$	$0.491$
$I$	$0.567$	$0.734$	$0.571$	$0.531$	$0.544$	$0.316$	$0.168$
$AI$	$0.316$	$0.371$	$0.320$	$0.203$	$0.316$	$0.762$	$0.762$

**Step 8.** The (normalized) values of each criterion are calculated with the help of Eq. (9), here ${C}_{1}$ to ${C}_{5}$ are benefit criteria, while ${C}_{6}$ to ${C}_{7}$ are cost criteria. Table 11 illustrates the standardized decision matrix of CFF.

**Table 11:** Normalized CFF decision matrix.

	${C}_{1}$	${C}_{2}$	${C}_{3}$	${C}_{4}$	${C}_{5}$	${C}_{6}$	${C}_{7}$
${A}_{1}$	$0.915$	$0.708$	$0.903$	$0.382$	$1.000$	$0.415$	$0.533$
${A}_{2}$	$0.557$	$0.584$	$0.909$	$0.973$	$0.980$	$0.615$	$0.526$
${A}_{3}$	$1.000$	$1.000$	$0.724$	$0.829$	$0.761$	$0.574$	$1.000$
${A}_{4}$	$0.936$	$0.614$	$0.560$	$0.978$	$0.581$	$0.659$	$0.221$
${A}_{5}$	$0.916$	$0.614$	$1.000$	$1.000$	$0.825$	$1.000$	$0.393$
${A}_{6}$	$0.746$	$0.564$	$0.929$	$1.000$	$0.968$	$0.608$	$0.317$
${A}_{7}$	$0.627$	$0.506$	$0.904$	$0.609$	$0.855$	$0.608$	$0.343$
$I$	$1.000$	$1.000$	$1.000$	$1.000$	$1.000$	$1.000$	$1.000$
$AI$	$0.557$	$0.506$	$0.560$	$0.382$	$0.581$	$0.415$	$0.221$

**Step 9.** The weighted values of the current alternative are calculated in (10). Tables 7 and 11 are also considered to get these weighted values. Table 12 is the weighted CFF decision matrix.

**Table 12:** Weighted CFF decision matrix.

	${C}_{1}$	${C}_{2}$	${C}_{3}$	${C}_{4}$	${C}_{5}$	${C}_{6}$	${C}_{7}$
${A}_{1}$	$0.139$	$0.108$	$0.134$	$0.053$	$0.136$	$0.056$	$0.072$
${A}_{2}$	$0.085$	$0.089$	$0.135$	$0.136$	$0.133$	$0.083$	$0.071$
${A}_{3}$	$0.152$	$0.152$	$0.108$	$0.116$	$0.103$	$0.078$	$0.136$
${A}_{4}$	$0.143$	$0.093$	$0.083$	$0.137$	$0.079$	$0.089$	$0.030$
${A}_{5}$	$0.140$	$0.093$	$0.149$	$0.140$	$0.112$	$0.136$	$0.053$
${A}_{6}$	$0.114$	$0.086$	$0.138$	$0.140$	$0.131$	$0.083$	$0.043$
${A}_{7}$	$0.095$	$0.077$	$0.134$	$0.085$	$0.116$	$0.083$	$0.047$
$I$	$0.152$	$0.152$	$0.149$	$0.140$	$0.136$	$0.136$	$0.136$
$AI$	$0.085$	$0.077$	$0.083$	$0.053$	$0.079$	$0.056$	$0.030$

**Step 10.** In this step, the $\breve{\d{{\text{\.{S}}}}}_{i}$ value of all of the alternatives will be calculated with the help of Eq. (11). In this way, $\breve{\d{{\text{\.{S}}}}}_{i}$ values obtained are presented in column 1 of Table 13.

**Table 13:** Utility degrees and utility functions of alternatives.

	${\d{{{\.{\text{S}}}}}}_{i}$	${\breve{{\mathfrak{K}}}}_{i}^{-}$	${\breve{{\mathfrak{K}}}}_{i}^{+}$	$f({\breve{{\mathfrak{K}}}}_{i}^{-})$	$f({\breve{{\mathfrak{K}}}}_{i}^{+})$	$f({\breve{{\mathfrak{K}}}}_{i})$	Rank
${A}_{1}$	$0.699$	$1.507$	$0.699$	$0.317$	$0.683$	$0.609$	$5$
${A}_{2}$	$0.733$	$1.580$	$0.733$	$0.317$	$0.683$	$0.639$	$4$
${A}_{3}$	$0.845$	$1.822$	$0.845$	$0.317$	$0.683$	$0.737$	$1$
${A}_{4}$	$0.654$	$1.411$	$0.654$	$0.317$	$0.683$	$0.570$	$6$
${A}_{5}$	$0.822$	$1.773$	$0.822$	$0.317$	$0.683$	$0.717$	$2$
${A}_{6}$	$0.734$	$1.583$	$0.734$	$0.317$	$0.683$	$0.640$	$3$
${A}_{7}$	$0.637$	$1.374$	$0.637$	$0.317$	$0.683$	$0.555$	$7$

**Step 11.** Utility degree of alternatives is calculated on (12) and (13). Therefore, the second and third columns of Table 13 show the alternatives’ degrees of utility.

**Step 12.** To obtain the utility functions of alternatives, the utility functions based on ideal and anti-ideal solutions should be calculated at first using Eqs. (15) and (16). Then, with the help of Eq. (14), the utility functions of alternatives are determined. Consequently, Table 13 shows an ideal solution, an anti-ideal solution, and the utility of other options considered.

**Step 13.** Finally, the ranking of alternatives follows in the order of their utility functions.

### Result discussion

**Fig. 2:**
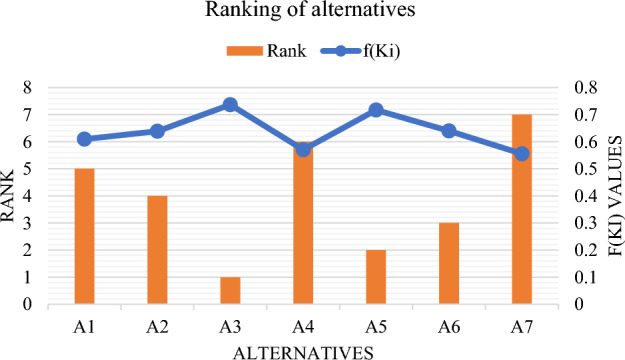
Ranking of the alternatives.

The results generated using the proposed CFF-MARCOS method provide a comprehensive ranking of seven deep learning models for dance classification. As shown in Table 13 and visualized in Fig. 2, model $A_{5}$ ranked highest, reflecting its superior performance in accuracy, precision, F1-score, generalization, and computational efficiency. This outcome confirms that the CFF-MARCOS-based ranking aligns with standard accuracy-based selection when one model clearly outperforms others. However, the framework also reveals trade-offs that may be overlooked by accuracy alone. As an example, ${A}_{4}$ is ranked second since it has a lower inference time and a simpler structure; thus, it can be deployed as a real-time application, and its accuracy is slightly lower than that of ${A}_{5}$. Model ${A}_{1}$ was in the third position, and it demonstrated good predictive metrics; however, it still needed more training time and was more complex, which restricted its applications. ${A}_{6}$ and ${A}_{2}$ performed moderately, whereas ${A}_{3}$ and ${A}_{7}$ ones ranked low because of poor results in the speed and precision. Generally, the CFF-MARCOS method integrates both positive sides of quantitative and qualitative strengths of generic models with the help of expert-judged presence of multi-criteria reasoning. It confirms classical metrics where it is correct, but also provides a more detailed and situation-sensitive model selection strategy.

### Theoretical implications

The theoretical findings of the implementation of the CFF-MARCOS approach to the current study have immense consequences for the MCDM literature and deep learning-based classification. To this end, the study enhances MCDM theory by illustrating how a greater degree of expressiveness of CFFS (as well as the ability to express hesitation) can be applied in complex decision-making tasks involving uncertain, inaccurate, and subjective judgments. Furthermore, the proposed study addresses the gap between fuzzy MCDM theory and artificial intelligence by introducing a methodological approach for evaluating pretrained deep learning models, considering technical performance and computational efficiency. The results confirm the potential of the CFF-MARCOS method as a powerful decision support tool in AI implementation, particularly where evaluation standards are not purely quantitative and multiple experts participate in the decision-making process. This contribution opens new avenues for further theoretical research and developing mixed fuzzy decision sciences and intelligent systems. Additionally, the CFF-MARCOS approach demonstrates computational efficiency, with complexity scaling linearly concerning the number of alternatives, criteria, and decision-makers. This property makes the model suitable for large-scale and real-time decision-making environments. Such scalability and low computational cost enhance the model’s theoretical robustness and practical applicability.

## Sensitivity analysis

**Fig. 3:**
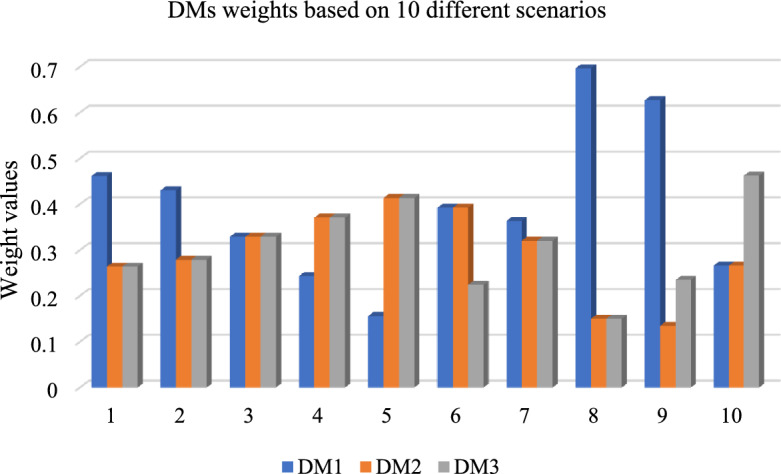
DM’s weights are based on ten different scenarios.

To determine the robustness, reliability, and effectiveness of the suggested CFF-MARCOS strategy, a sensitivity analysis was conducted by systematically varying the weights of decision-makers across ten different scenarios. In each instance, the weights assigned to $DM_{1}$, $DM_{2}$ and $DM_{3}$ were adjusted (as illustrated in Fig. 3) to simulate a variety of decision-making environments and capture subjective fluctuations in expert influence.

**Fig. 4:**
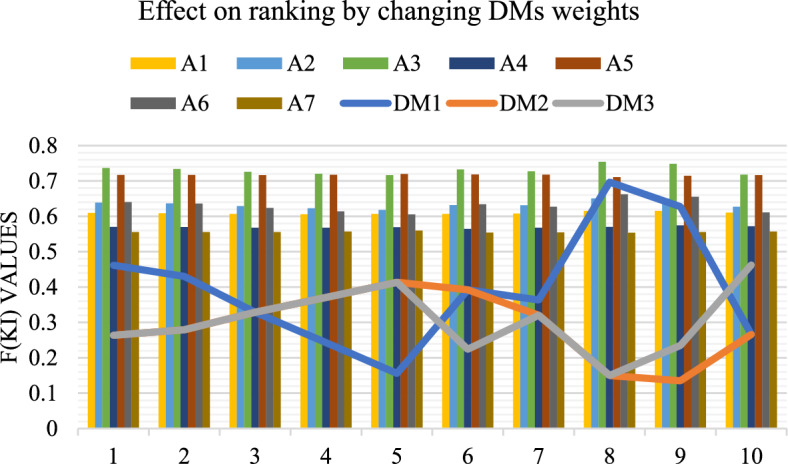
Effect on the ranking of alternatives based on DM’s weights.

The cumulative utility degrees for each alternative ($A_{1}$ to $A_{7}$) were then recalculated in each scenario. The results, shown in Fig. 4, provide a visual representation of how the rankings of alternatives responded to these changes. Notably, $A_{3}$ consistently maintained a top position, highlighting its strong classification capabilities and resilience to expert weight variation. Similarly, $A_{5}$ also demonstrated high consistency, retaining a favorable rank across nearly all test cases. On the other hand, alternatives such as $A_{7}$ and $A_{4}$ exhibited more sensitivity to expert weighting, occasionally shifting positions based on how the influence of decision-makers changed. These variations underline the importance of weight assignment but also emphasize the stability of top-performing models under the CFF-MARCOS framework. This analysis confirms that the final ranking outcomes are largely stable and reliable, even with moderate changes in decision-maker priorities. Therefore, the proposed method exhibits a high degree of decision robustness, validating the flexibility and credibility of the CFF-MARCOS approach in expert-driven model selection for dance classification tasks.

### Comparison with existing MCDM approaches

To demonstrate the effectiveness of the proposed CFF-MARCOS method, a comparison was made with five widely-used MCDM techniques: WASPAS, VIKOR, EDAS, COCOSO, and TOPSIS. The evaluation criteria included: handling of uncertainty, support for fuzzy environments, stability of results, sensitivity to criteria weights, computational efficiency, ease of implementation, result interpretability, and multi-expert decision support. Table 14 summarizes the comparative analysis. As shown, the CFF-MARCOS method consistently outperforms the others, particularly in modeling high-dimensional uncertainty and incorporating expert preferences using CFFS. While traditional methods often lack flexibility in managing such uncertainty, CFF-MARCOS provides more robust and interpretable outcomes, making it especially effective for real-world applications like dance classification using pretrained deep learning models.

**Table 14:** Comparison analysis*.*

Criteria	CFF-MARCOS	WASPAS^4^	VIKOR^5^	EDAS^6^	COCOSO^7^	TOPSIS^8^
Handling Uncertainty	High	Medium	Medium	Low	Medium	Low
Incorporation of CFFS	High	Low	Low	Low	Low	Low
Result Stability Across Scenarios	High	Medium	Medium	Low	Medium	Medium
Sensitivity to Criteria Weights	High	Medium	High	High	Medium	High
Computational Complexity	High	Medium	Medium	Medium	High	Medium
Ease of Implementation	High	High	Medium	High	Medium	High
Interpretability of Results	High	Medium	Medium	High	Medium	Medium
Multi-Expert Integration	High	Medium	Medium	Low	Medium	Low
Adaptability to Fuzzy Environments	High	Medium	Medium	Low	Medium	Low

### Advantages of the study

The suggested CFF-MARCOS approach offers several unique advantages over current methods of MCDM. First, it improves the decision-making process by incorporating a new level of modelling uncertainty according to CFFS, making it more flexible and expressive in capturing expert opinions, especially when available data is less precise or evaluated qualitatively. Second, the method supports decision-making involving multiple experts, ensuring that the integration of different opinions and estimations does not diminish their importance. Third, CFF-MARCOS strikes a considerable balance between computational fidelity and the interpretation of the patterns found in the ranking and selection processes, allowing decision-makers to follow and explain all operations carried out. The approach also provides many benefits and meets cost requirements without requiring transformations, thereby simplifying the evaluation structure. Additionally, the method has been recognized as sensitive and robust through sensitivity analyses, which suggests it delivers consistent results across various situations, even when the weights assigned by decision-makers are not equal. Finally, its feasibility and adaptability are demonstrated through real-life applications, such as classifying dances with the aid of pretrained deep learning models. All these advantages make CFF-MARCOS a valuable and relevant resource for contemporary decision-making.

### Managerial implications and limitations

The CFF-MARCOS technique outlined in the paper has several key managerial implications primarily aimed at individuals with backgrounds in artificial intelligence, decision sciences, and performing arts. By introducing pretrained deep learning models for dance classification within an effective MCDM framework, it enables stakeholders to manage systems in situations marked by uncertainty and conflicting demands. Specifically, it supports real-world applications by assisting managers in deciding which AI model to adopt, considering not only technical metrics but also subjective expert opinions, which are crucial in creative fields such as dance. CFFS provides nuanced flexibility in expressing expert judgments, thereby enhancing the reliability and validity of the model selection.

From a management perspective, this CFF-MARCOS framework communicates the computational assessment to human perception. For example, its relevant application could be in guiding project leaders involved in intelligent dance training or in automating performance evaluation to identify models that balance the trade-offs between performance analysis accuracy, inference time, and model complexity. This enables an informed choice of deployment aligned with system performance requirements and stakeholder preferences. Additionally, it promotes better cross-functional collaboration between developers, choreographers, and data scientists by illustrating the interactions among various performance indicators in multi-expert systems.

The method, however, has limitations, even though it has been adjusted to enhance its benefits. One major challenge is the computational complexity involved in processing CCFVs; this can be costly, especially with large files or in tasks requiring time-based decisions like model selection in real-time scenarios or streaming classification operations. It is also a complex process involving a series of matrices to determine decisions, normalization, utility, and the combining weights of professionals, which can be very tedious without macro-enabled programs or specialized toolkits. Using experts in assessment is another drawback; if inputs from decision-makers are inaccurate, limited, or too vague, it can negatively affect the outcome. Also, managers unfamiliar with fuzzy set theory or more complex models of MCDM will be presented with a significant learning curve, and this is unlikely to occur easily in non-technical disciplines without special visualization or interpretability modules.

## Conclusion

In this paper, an approach based on the combination of pretrained deep learning models and CFF-MARCOS is proposed to evaluate and rank dance-classification models under uncertainty. The findings confirm the effectiveness of the method to control how the subjective evaluation of experts is done and the explosion of multiple performance requirements. Stability of the ranking was confirmed with sensitivity analysis, and with comparative analysis revealing that the method is superior to conventional methodologies of MCDM. These results prove the effectiveness, dependability, and feasibility of the model to be utilized in an actual scenario of AI-powered systems. Besides its applicability to make informed decisions regarding dance classification, the method has the advantage of providing generalizability to other disciplines where careful and uncertain assessments are essential, such as health care, automation, and financial methods.

Future work should focus on simplifying computational complexity and could be directed at extending the proposed CFF-MARCOS approach based on other advanced fuzzy environments, including spherical fuzzy sets^34^ and interval-valued picture fuzzy sets with Frank operators^35^. One can also enhance decision accuracy through the integration of complex fuzzy models such as Dombi aggregation^36^ and prioritized Muirhead means under neutrosophic environments^37^. Interval-valued T-spherical fuzzy system-based^38^ techniques, similar to MCDM, also hold significant promise. Additionally, the practical usefulness of this work can be expanded by adapting circular fuzzy approaches like the CIF-EDAS to automotive decision-making^39^ and other related fields.

## Data Availability

The datasets used and/or analyzed during the current study are available from the corresponding author on reasonable request.

## Abbreviations

CFFS: Circular Fermatean fuzzy sets
MARCOS: Measurement alternatives and ranking according to compromise solution
CFF-MARCOS: Circular Fermatean fuzzy MARCOS
MCDM: Multi-criteria decision making
DM: Decision maker
AI: Artificial intelligence
DL: Deep learning
CNNs: Convolutional neural networks
TOPSIS: Technique for order preference by similarity to ideal solution
VIKOR: VlseKriterijumska Optimizacija I Kompromisno Resenje
EDAS: Evaluation based on distance from average solution
COCOSO: Combined compromise solution
WASPAS: Weighted aggregated sum product assessment
MD: Membership degree
NMD: Non-membership degree
HD: Hesitance degree

## List of symbols

$$\dot{\text{X}}$$: Universal set
$x$: Element of sets
$$\dot{\text{H}}$$: CFFS
$$\mathfrak{p}$$: MD
$$\mathfrak{q}$$: NMD
$$\mathcalligra{h}$$: HD
$$\underset{\raise0.3em\hbox{$\smash{\scriptscriptstyle\cdot}$}}{\text{r}}$$: Radius term
$D$: Set of DMs
$\mathcalligra{w}$: Weights of DMs
$\mathcal{W}$: Weights of criteria
$\mathcal{G}$: CFF decision matrix
$$\tau$$: Ideal solutions
$$\mathcal{D}$$: Distance measures
$$CC$$: Closeness coefficients
$$\breve{\mathfrak{K}}$$: Utility degree
$$f({\mathfrak{K}}_{i})$$: Utility function
$$A$$: Alternative
$$C$$: Criteria
